# Paraneoplastic Evans Syndrome in a Patient With Prostate Cancer With Small Cell Transformation

**DOI:** 10.7759/cureus.24505

**Published:** 2022-04-26

**Authors:** Adarsh Sidda, Gurusidda Manu, Mahmoud Abdallah, Doreen Griswold, Mohamed Alsharedi, Toni Pacioles

**Affiliations:** 1 Hematology and Medical Oncology, Marshall University Joan C. Edwards School of Medicine, Huntington, USA; 2 Internal Medicine, University of Texas Southwestern Medical Center, Dallas, USA; 3 Internal Medicine, Marshall University Joan C. Edwards School of Medicine, Huntington, USA; 4 Pathology, Marshall University Joan C. Edwards School of Medicine, Huntington, USA; 5 Oncology/Hematology-Oncology, Marshall University Joan C. Edwards School of Medicine, Huntington, USA; 6 Hematology and Oncology, Marshall University Joan C. Edwards School of Medicine, Huntington, USA

**Keywords:** evans syndrome, immune-mediated thrombocytopenia, prostate cancer, small cell carcinoma of the prostate, paraneopastic syndrome, autoimmune hemolytic anemia (aiha)

## Abstract

Paraneoplastic syndromes are defined as tumor‐associated indirect systemic effects. Prostate cancer-associated paraneoplastic syndromes typically have endocrine, neurologic, and dermatologic manifestations. Reports have suggested up to 70% of metastatic prostate cancers manifest as paraneoplastic entities. Although common in hematological malignancies, it is rare for prostate cancer and other solid tumors to be associated with immune-mediated cytopenias such as Evans syndrome. Based on our PubMed search for the keywords Evans syndrome and prostate cancer, only one other case has been reported in the literature with this association. We report the second such case in a 63-year-old male who initially presented with hemolytic anemia and thrombocytopenia. He was diagnosed with Evans syndrome with initial responses to both steroids and intravenous immunoglobulin. Extensive workup, including an eventual bone marrow biopsy, revealed metastatic prostate cancer with transformation to small cell neuroendocrine carcinoma.

## Introduction

Paraneoplastic syndromes (PNS) are defined as tumor‐associated indirect systemic effects. Paraneoplastic hematologic syndromes (PHS), although rare, have been associated with solid tumors in the past. Autoimmune hemolytic anemia (AIHA) is a well-known paraneoplastic phenomenon in lymphoproliferative disorders. AIHA in combination with immune-mediated thrombocytopenia is commonly known as Evans syndrome. We present a rare case of paraneoplastic syndrome manifesting as Evans syndrome in a patient with advanced transformed small cell carcinoma of the prostate.

## Case presentation

A 63-year-old male patient with hypertension, remote history of deep venous thrombosis, obesity, and tobacco dependence initially presented to the hospital with dysuria and urinary obstruction. CT abdomen and pelvis on admission showed bilateral hydronephrosis with multiple peri-aortic lymph nodes. It also showed an enlarged prostate with a soft tissue mass extending posteriorly into the bladder and measuring 6.0 x 4.5 cm. His prostate-specific antigen (PSA) level was 73. Transrectal prostate biopsy and cystoscopy were done. The biopsy result was consistent with prostatic adenocarcinoma with a Gleason score of 5+5=10. Subsequent MRI of the spine, as well as a bone scan, showed diffuse skeletal metastasis involving the lumbosacral vertebrae consistent with metastatic disease. The patient was started on abiraterone + prednisone along with androgen deprivation therapy of leuprorelin every six months. The patient responded very well to the treatment with a PSA nadir of <0.01 and testosterone levels of <10.

Six months after the initial visit, the patient returned to the emergency room (ER) with fatigue. Lab values were significant for new normocytic anemia of 7 g/dL and thrombocytopenia with a platelet count of 34,000. Both values were in the normal range a month prior to this presentation.

CT chest revealed a pulmonary embolism with no evidence of right ventricular strain. Anticoagulation was not initiated due to his thrombocytopenia. He underwent an inferior vena cava filter placement. A thorough workup of his anemia showed elevated lactate dehydrogenase levels, undetectable haptoglobin, high reticulocyte count, and elevated indirect bilirubin. The peripheral smear showed thrombocytopenia and normocytic anemia with no evidence of schistocytes. Other types of workup, including AdamTS-13, cold agglutinins, and paroxysmal nocturnal hemoglobinuria (PNH), were all negative. Coombs test was also interestingly negative. The patient was started on intravenous (IV) steroids with methylprednisone for suspected hemolytic anemia and immune thrombocytopenia (ITP), thought to be from Evans syndrome. He showed an initial response to the steroids with improved hemoglobin and platelet counts as well as decreasing lactate dehydrogenase (LDH), indirect bilirubin, and increasing haptoglobin. Eventually, he proved refractory to IV steroids and was started on a trial of intravenous (IV) immunoglobulin (IVIG) therapy. Patients responded to this treatment with improved hemoglobin and platelet counts but proved refractory to IVIG as well. We eventually pursued a bone marrow aspirate and biopsy for further investigation.

**Figure 1:**
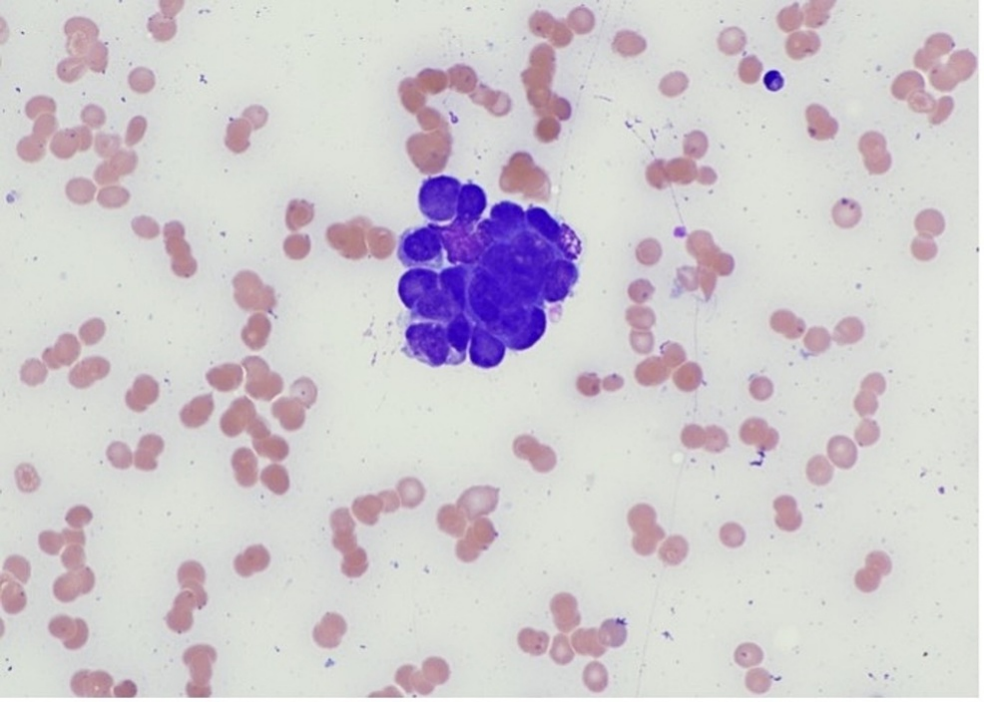
Bone marrow aspirate smear showing a cohesive group of non-hematopoietic malignant cells Photographed at 50 x oil; Wright Giemsa stain

**Figure 2:**
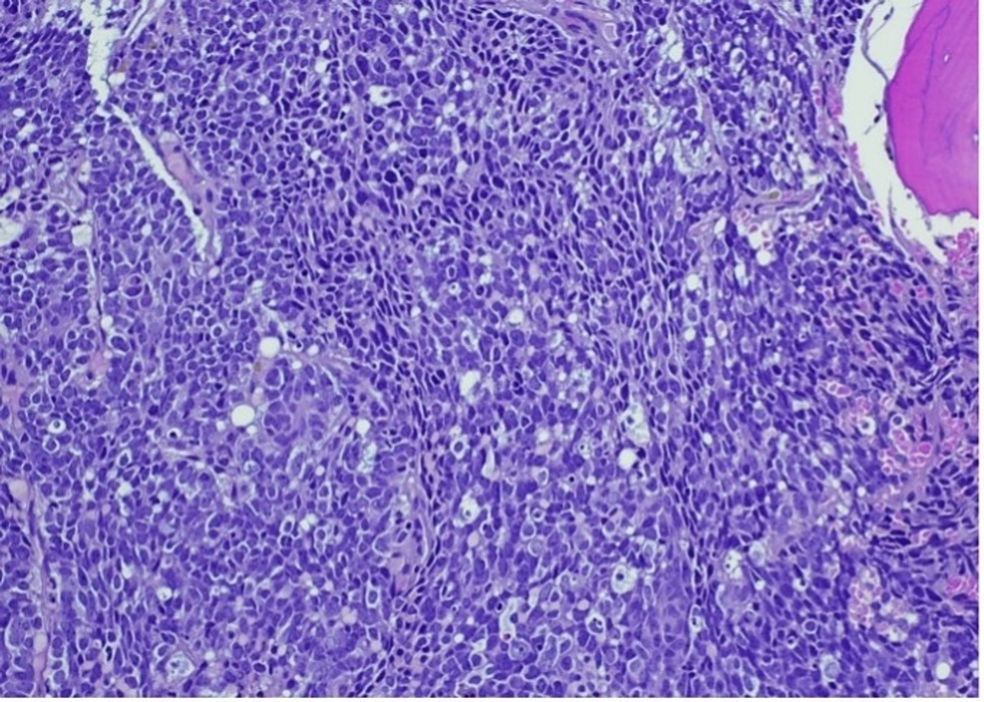
Bone marrow core biopsy showing an infiltrate of malignant, cohesive cells with areas of nuclear molding Photographed at 20 x; hematoxylin & eosin stain

**Figure 3:**
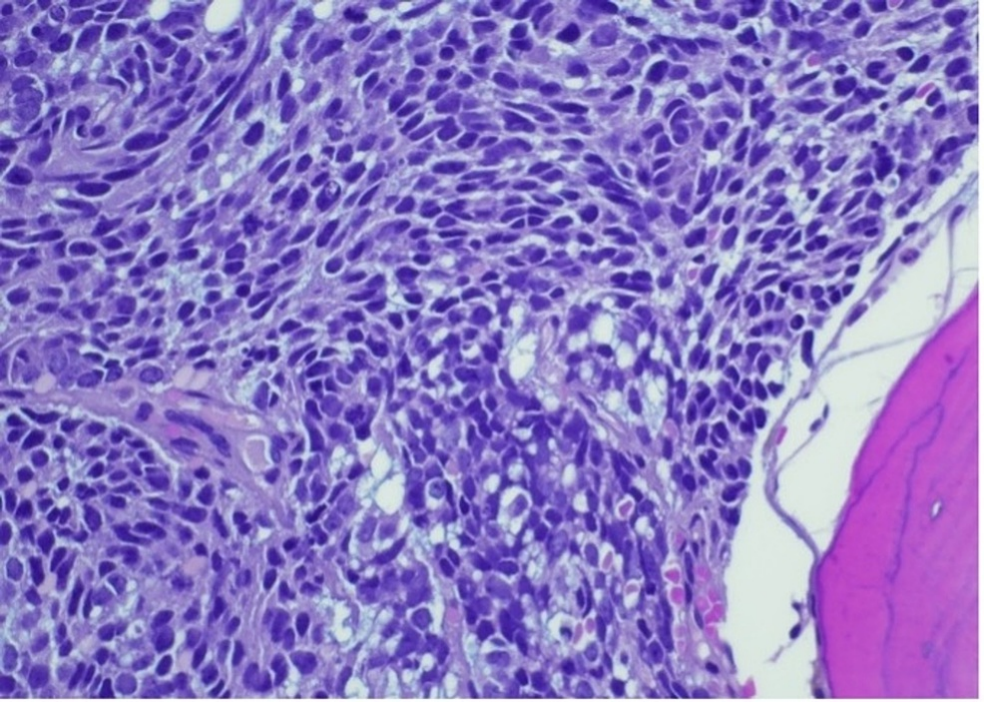
Bone marrow core biopsy showing nuclear detail of finely stippled nuclear chromatin and small chromocenters. Photographed at 40 x; hematoxylin & eosin stain

**Figure 4:**
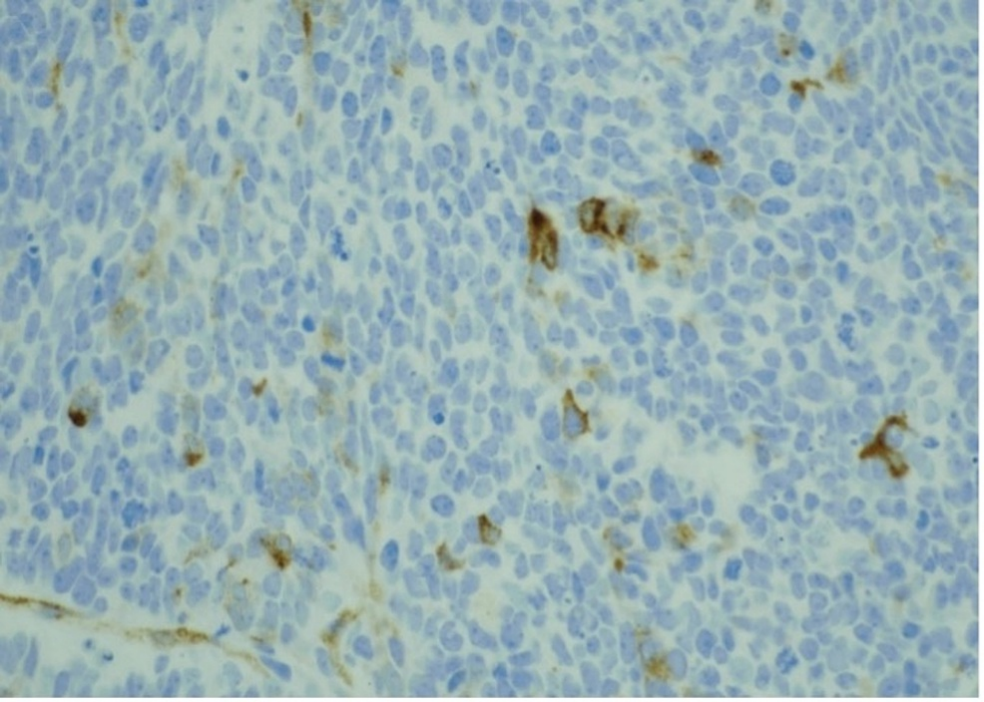
Bone marrow core biopsy stained with prostate-specific membrane antigen (PSMA) showing occasional positive cells Photographed at 40 x

**Figure 5:**
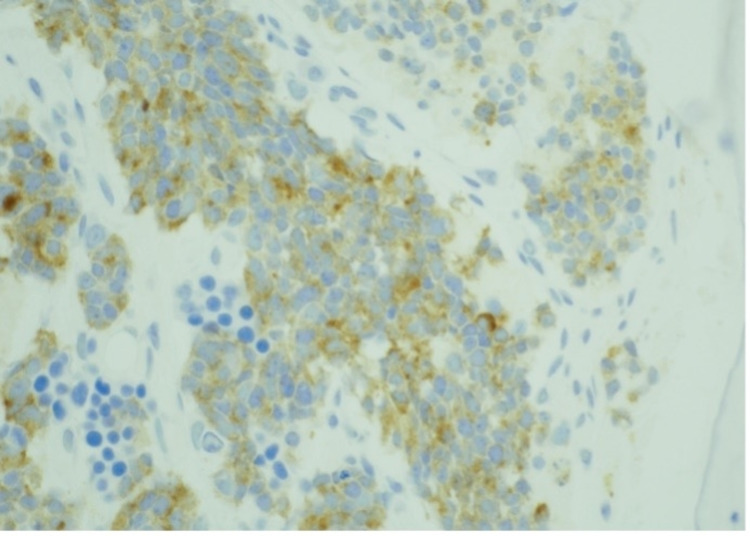
Bone marrow core biopsy stained with synaptophysin showing positive expression Photographed at 40 x

**Figure 6:**
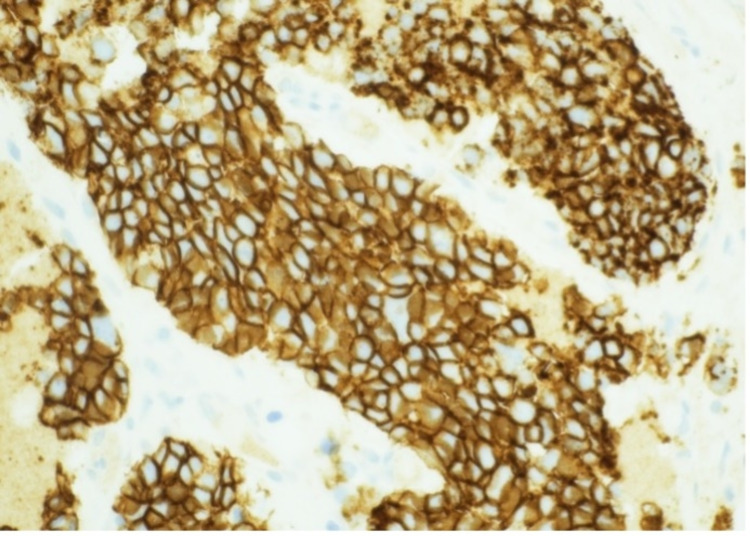
Bone marrow core biopsy of CD 56 Photographed at 40 x

Bone marrow revealed numerous non-hematopoietic malignant cells in the aspirate specimen (Figure 1). Core biopsy showed an infiltrate of malignant cells with nuclear molding (Figures 2-3). Prostate-specific membrane antigen (PSMA) stain confirmed prostatic origin (Figure 4). Synaptophysin and CD56 were also positive (Figures 5-6) indicating transformation to neuroendocrine/small cell carcinoma [1]. Considerations were given to start the patient on carboplatin/etoposide but given his poor performance status and extended discussion regarding his goals of care, he was made comfort care and passed away a few days later.

## Discussion

Paraneoplastic syndrome is defined as findings that arise from the indirect effect of tumors that cannot be attributed to tumor invasion or metastasis. They are typically associated with hematological malignancies, such as lymphomas and leukemia [2], and are thought to be caused by the secretion of cytokines, hormones, or peptides and involve immune cross-reactivity between normal and malignant tissue [3].

Various paraneoplastic syndromes that have been reported to be associated with prostate cancer are SAPHO (synovitis, acne, pustulosis, hyperostosis, and osteitis) syndrome [4], hyperfibrinolysis [5], giant cell arteritis [6], Cushing syndrome [7], Stauffer syndrome [8], anti-Hu mediated intestinal pseudo-obstruction [9], and SIADH (syndrome of inappropriate antidiuretic hormone secretion) [10].

Evans syndrome (ES) is usually characterized by the co-occurrence of two or more immune cytopenias, most commonly AIHA and thrombocytopenia. It was defined by Robert Evans in 1951 [11], and since its first description, ES has long been considered as a rather incidental and “anecdotal” combination of ITP and AIHA and/or autoimmune neutropenia in the absence of any underlying cause. More recently, the spectrum of the disease has broadened to reflect a state of profound immune dysregulation as opposed to a coincidental combination of immune cytopenias.

At a genetic level, mutations that are linked to Evans syndrome are found in autoimmune lymphoproliferative syndrome (ALPS)-FAS gene, cytotoxic T lymphocyte antigen-4 (CTLA-4), and lipopolysaccharide-responsive vesicle trafficking beige-like and anchor protein (LRBA) [12]. These could be potential etiologies for the development of Evans syndrome.

Evans syndrome typically presents with anemia, thrombocytopenia, and a positive Coombs test. Our patient presented with all such parameters except for a negative Coombs test. There are data that suggest that up to 10% of Evans syndrome can present with a negative DAT [13]. Being Coombs negative made this case a diagnostic challenge but initial responsiveness to steroid and IVIG treatments was strongly supportive of an immunologic process. Biopsy confirmation of transformation to small cell carcinoma and its strong association with numerous paraneoplastic processes strengthened our hypothesis.

Evans syndrome is mainly treated with a combination of glucocorticoid and intravenous gamma globulin in the acute setting. Rituximab and/or mycophenolate mofetil are often employed after initial response or in refractory settings. Evans syndrome is overall considered difficult to treat, as it is typically less responsive to standard therapies with frequent relapses and higher mortality than isolated warm AIHA [14]. In a series of 68 patients with Evans syndrome, short-term responses were seen in over 80%, but only 22 (32%) were in remission off treatment at a median follow-up of 4.8 years and 16 (24 percent) had died.

Small cell transformation of prostate cancer is rare and oftentimes fatal. These tumors show positivity for neuroendocrine markers such as chromogranin, synaptophysin, and CD 56 [15]. Only half of these small-cell neuroendocrine tumors express the TTF-1 marker. Prostate-specific markers (PSA, PSAP, PSMA, and NKX3.1) may also be lost or only partially expressed. These tumors are typically treated with platinum-based chemotherapy similar to pulmonary small cell tumors, as they are often resistant to androgen deprivation therapy [16].

Autoimmune cytopenias appear to be relatively common in cancers such as lymphomas [17] and leukemias while it is very rare in solid tumors such as the breast, pancreas, and prostate [18]. It is important to recognize the small-cell transformation potential in patients with prostate cancer and to also be vigilant of its various paraneoplastic entities [19]. It is also important to once again recognize that up to 10% of the cases of Evans syndrome may manifest with a negative direct antiglobulin test (DAT). This is only the second reported case of Evans syndrome presenting in a patient with prostate cancer with the first case reported in Japan in 2016 [20]. 

## Conclusions

The discovery of various paraneoplastic manifestations in solid tumors continues to grow. Evans syndrome is an autoimmune-mediated process that should be suspected in patients with hemolytic anemia with concurrent immune-mediated thrombocytopenia. Diagnostic challenges may include DAT-negative hemolysis. Management of this disease can also be challenging, as the disease is oftentimes refractory to currently recommended treatments. Data are still lacking with regard to adequate management options. Although Evans syndrome and paraneoplastic processes are rare, being aware of these paraneoplastic associations is important for the prompt recognition and management of these patients in clinical practice.
